# The Hospital World

**Published:** 1910-08-20

**Authors:** 


### The Hospital World.

### Personalia.

Miss A. L. Burleigh, who came from England nine years ago to be matron of the Melbourne Hospital, has resigned her position from October 10 next.

Mr. Bell, in seconding the report at the annual general meeting of the Belfast Hospital for Diseases of the Skin, referred to the loss the hospital had sustained by the death of Canon Scott, whose time and advice were always at the committee's disposal, and stated that he was sure they all deeply sympathised with their president, Mr. E. W. Pim, J.P., in the great loss he had sustained in the death of his wife.

Mr. W. H. Moore, of the secretarial staff of the Westminster Hospital, has been appointed secretary-superintendent of the Lincoln County Hospital. Mr. W. B. Danby, who has been secretary of the institution for nearly thirty years, has resigned, severing thus a family connection with the hospital, which was started by his father, who was secretary for thirty-six years. Another retirement is that of Mr. W. Watkins, who for many years has been a member of the surgical staff.

At the annual meeting of subscribers to Totnes Cottage Hospital, the Duke of Somerset was re-elected patron; Mr. A. M. Singer, president; Mr. C. Barran, hon. sec.; Mr. J. Todd, assistant secretary; and Mr. F. Telfer, treasurer to the hospital. The committees were re-elected, Mrs. A. Michelmore and Mrs. A. M. Champernowne were invited to act on the Ladies' Committee, and Drs. Allingham and Jellicoe were added to the medical staff.

We have to record with regret the death of Mr. Owen E. Hayter, chairman of the committee of the Cancer Hospital (Free), Brompton, S.W. Mr. Hayter joined the board in 1901, and was elected chairman in 1906. During his term of office important additions were made to the hospital, and he showed himself a most energetic and capable chairman, always working for the good of the institution. The funeral took place on Thursday, August 11, a service being held in St. Augustine's Church, Queen's Gate, the interment being in the family vault at Brompton Cemetery. Among those present were Messrs. Campbell Cooper and F. L. Chattell, representing the house committee; Messrs. Leaf and Swan, representing the medical staff; Dr. Alexander Paine, director Research Institute; Mr. A. M. Hooper, assistant secretary, on behalf of the secretary, absent on account of illness; Mr. Alex. Graham, architect; and also many members of the nursing and domestic staff. Among the numerous floral tokens were those of the President, the Earl of Northbrook; the House Committee; Medical Staff; Secretary; Assistant Secretary and Clerk; Matron; Sisters Mossman and Cort; Nursing Staff and Domestic Staff.

A very graceful compliment was paid the other day to Mr. Arthur Hill, Hon. Treasurer of the Hull Victoria Hospital for Sick Children, when Mr. Grant (the Chairman) presented him with a silver snuffbox. The House Committee, we understand, met as usual, when Mr. Grant surprised Mr. Hill by reminding the meeting that for twenty-five years Mr. Hill had been a member of the Committee, during which time his enthusiasm had been constant, and his work quiet and constant too. In the spring of 1885 Mr. Hill was elected by the members on the governing body, and four years later he became Hon. Assistant Treasurer. Mr. Francis Pease was Treasurer at that time, and held the office till June 1907, when he was succeeded by Mr. Hill. This is a record of work of which the hospital and Mr. Hill himself must be proud, and the presentation was peculiarly appropriate because Mr. Grant too is an old and tried worker, who knows from experience what the value of the work is which the meeting was delighting to honour. Tradition is formed only by the occupation of one place over a period of years by the same group of people, and a hospital which keeps its officers unchanged is building up a tradition which will influence its life for good in a thousand ways. May Mr. Hill hold his office for many years to consolidate this tradition still further!

Dr. William Alexander has just retired from the position of visiting surgeon to the Liverpool Workhouse Hospital, with which institution he has been associated for nearly forty years. His resignation was received with much regret by the Select Vestry at their meeting last week, and a warm tribute was paid to his long and valuable services by some of the members. A resolution of appreciation of his services was proposed by Mr. T. D. Laurence, and seconded by Dr. Follows. Mr. Edward Horrigan, in supporting, said that Dr. Alexander, when he entered the service of the Vestry nearly forty years ago, was a young, unknown Irish doctor, but by his ability, conscientiousness, and strong sense of duty he had risen step by step from resident medical officer to district medical officer, and then to visiting surgeon, until at the present time, and for many years past, he enjoyed the reputation of being one of the foremost surgeons in the North of England. Few outside the sphere of Poor-law work had any conception of the nature, extent, and complexity of the work which Dr. Alexander was called upon to perform. When he (Mr. Horrigan) mentioned that for many years on the surgical side of the hospital Dr. Alexander had to treat nearly 9,000 cases per annum, some idea of the magnitude and responsibility of his duties might be realised; and the total mortality and death-rate was the low figure of 51 per 1,000 living—a powerful testimony of the skill and care with which the treatment was carried out. Their hospital had, during recent years, been subjected to much searching criticism with regard to its structure and appointments, the most flattering terms employed being "old," "obsolete," and "antiquated"; but notwithstanding these material defects, under the supervision of Dr. Alexander the results attained would not only compare favourably with other institutions, but in many instances far excelled anything accomplished in many of our modern and perfectly equipped hospitals.

